# Duodenal Perforation in the Setting of Retrievable Inferior Vena Cava Filter

**DOI:** 10.7759/cureus.86712

**Published:** 2025-06-25

**Authors:** Tyler Liguori, Oleg Orlov, Christian Gutierrez, John Danks

**Affiliations:** 1 Surgery, Carepoint Health, Jersey City, USA; 2 Surgery, St. George's University School of Medicine, St. George's, GRD; 3 Vascular Surgery, St Joseph University Medical Center, Paterson, USA

**Keywords:** acute pulmonary embolism, intestinal perforation, ivc filter complication, ivc filter migration, ivc filter placement

## Abstract

Inferior vena cava filters are medical devices used as an alternative method in the prevention of pulmonary thromboembolism in patients with deep vein thrombosis. Though generally regarded as safe alternatives, these devices do carry a risk of associated complications. Presented is a case of a 32-year-old female who developed a duodenal perforation after undergoing the removal of a retrievable inferior vena cava (IVC) filter. She required placement of the IVC filter during her second pregnancy due to multiple thrombotic events. The patient underwent nonoperative conservative therapy and ultimately had a favorable outcome. When managing patients with known IVC filter placement experiencing acute-onset abdominal pain, there should be a high clinical suspicion of IVC filter perforation of an organ.

## Introduction

Inferior vena cava filters are devices utilised in the prevention of pulmonary embolism (PE) in patients with a risk of developing venous thromboembolism (VTE) or similar coagulopathies. Typically, IVC filters are reserved for patients who cannot tolerate or have an absolute contraindication to anticoagulation therapy [1]. These contraindications include patients with uncontrolled bleeds, active gastrointestinal and intracerebral bleeds, those at risk for major bleeding, such as individuals with coagulation defects or severe thrombocytopenia, hemorrhagic stroke, and patients in need of emergent surgery [2].

Though IVC filters are typically considered safe alternatives, some complications are associated with using these devices, which can be insertion-related or delayed. Perioperative complications associated with filter insertion include bleeding from the access site, thrombosis, infection, pneumothorax, filter tilt, filter misplacement, and incomplete opening [3]. Delayed complications include pulmonary embolism, thrombosis, filter migration, device fracture, vessel injury, and organ perforation [4]. Longer indwelling times, where the filters are subjected to increased periods of mechanical stress, have resulted in a significant number of complications, leading to decreased benefit-to-risk ratio when the filter is left in place for an extended time [1]. Therefore, IVC filter retrieval must be completed as soon as PE prophylaxis is no longer indicated.

Some filters may be left in place permanently, while others are designed to be removed after a period of time [1]. Most modern filters are designed to be retrievable; however, studies have found retrieval rates to range from 12% to 45%, meaning most filters become permanent devices [5]. The optimal window for retrieval can vary substantially between different IVC filter models. A systematic review found the average time to retrieval was 72 days, although studies have shown device-related complications to be highest in filters left in place for greater than 30 days [2].

This article was previously presented as a meeting abstract at the 2023 Eastern Vascular Society Annual Meeting.

## Case presentation

This is a case of a 32-year-old female with a past medical history of multiple deep venous thromboses (DVT) and pulmonary embolisms, requiring IVC filter placement five months prior, who presented to the emergency department due to right lower extremity swelling. The patient had a new onset of multiple pulmonary embolisms and deep venous thromboses during her second pregnancy, causing her to have a miscarriage two months prior to this presentation.

**Figure 1:**
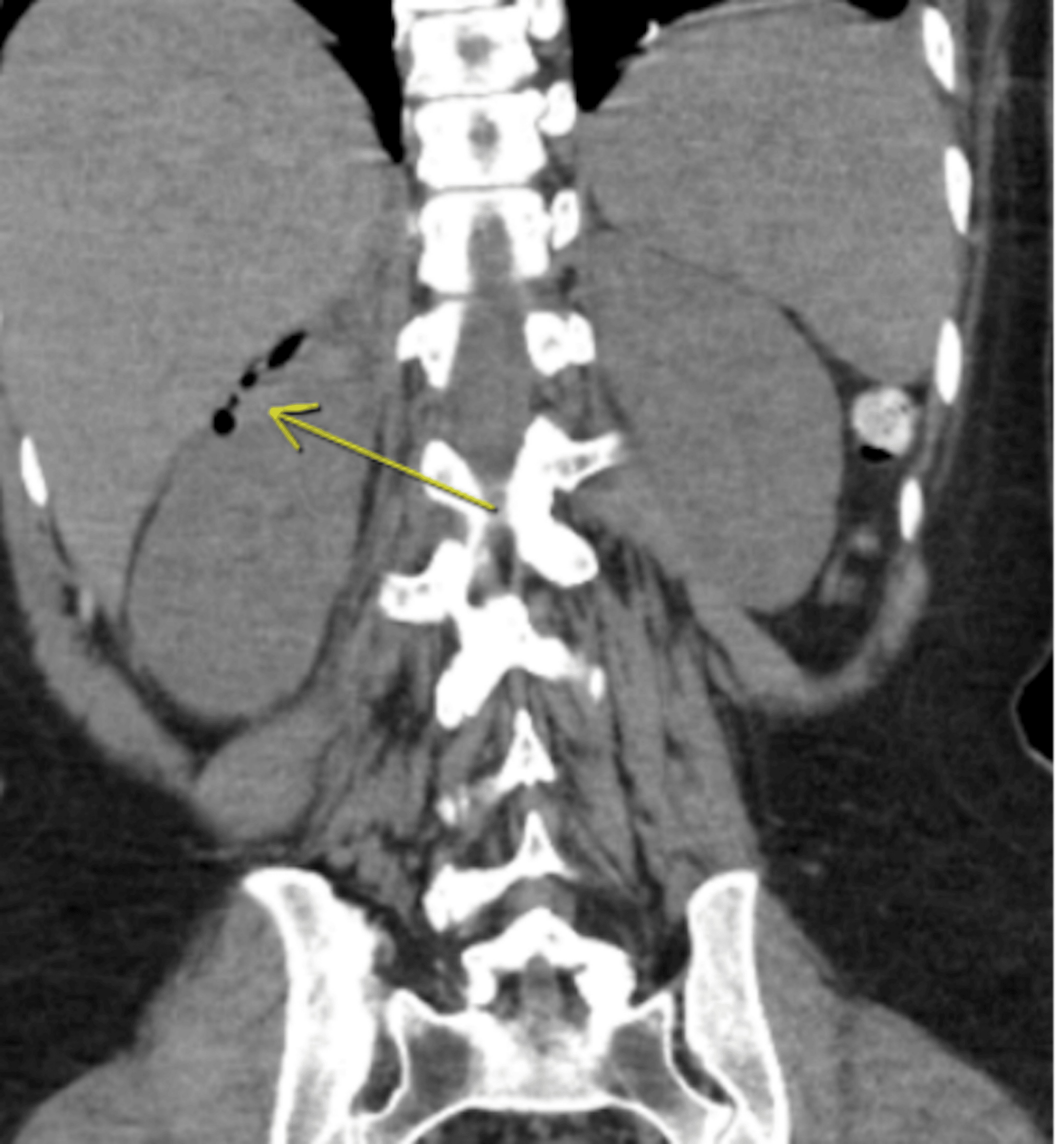
CT abdomen/pelvis coronal view showing subcutaneous emphysema in the right perirenal space.

The patient was started on a heparin drip, according to hospital protocol by weight and anti-Xa levels, with subsequent removal of the IVC filter and mechanical thrombectomy with no initial complications. A computed tomography (CT) taken three days after the procedure showed a metallic residual fragment in the inferior vena cava near the right renal vein, which correlated to incomplete IVC filter removal (Figure 1).

**Figure 2:**
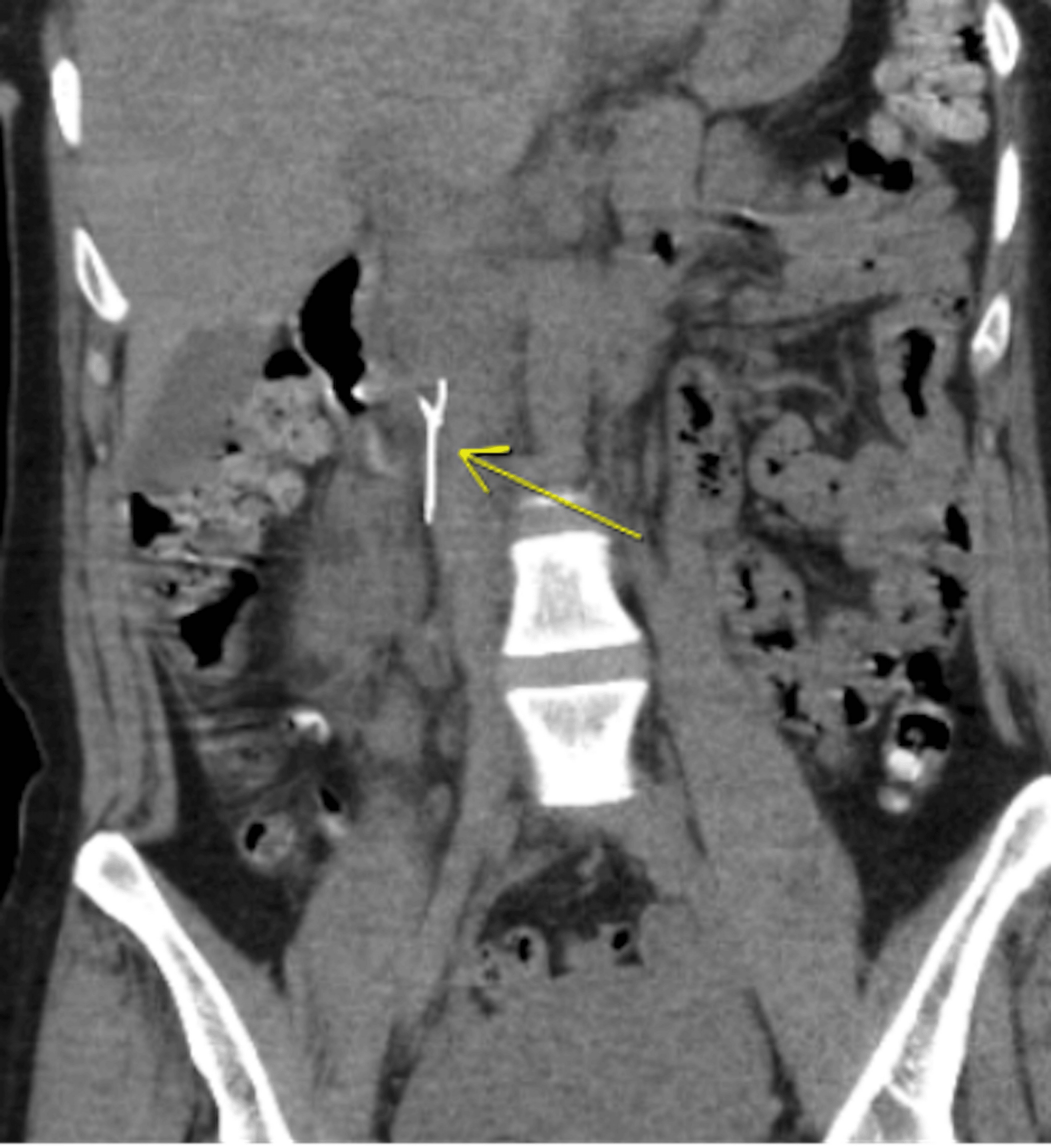
CT abdomen displaying linear metallic densities, consistent with retained IVC filter fragment.

Four days after the first procedure, she underwent another thrombectomy of her IVC and right external iliac vein. A second attempt was made to remove the residual filter fragment using a sweeping technique with an inflated balloon, but the attempts were unsuccessful. After this second procedure, the patient complained of persistent right-sided abdominal pain. A CT scan was obtained two days after the second procedure, showing extensive thrombus burden and air and hematoma in the retroperitoneum around the right kidney (Figure 2). The presence of gas within the retroperitoneum was concerning for a duodenal injury. Her hemoglobin dropped from the preoperative value of 13 to 10 grams per deciliter. Heparin was discontinued for one day, but with stable values of serial hemoglobin, she was restarted on a heparin drip. No further surgical interventions were indicated at the time. The patient was monitored closely for these signs, with the understanding that there was a possibility of operative intervention if conservative management failed.

**Figure 3:**
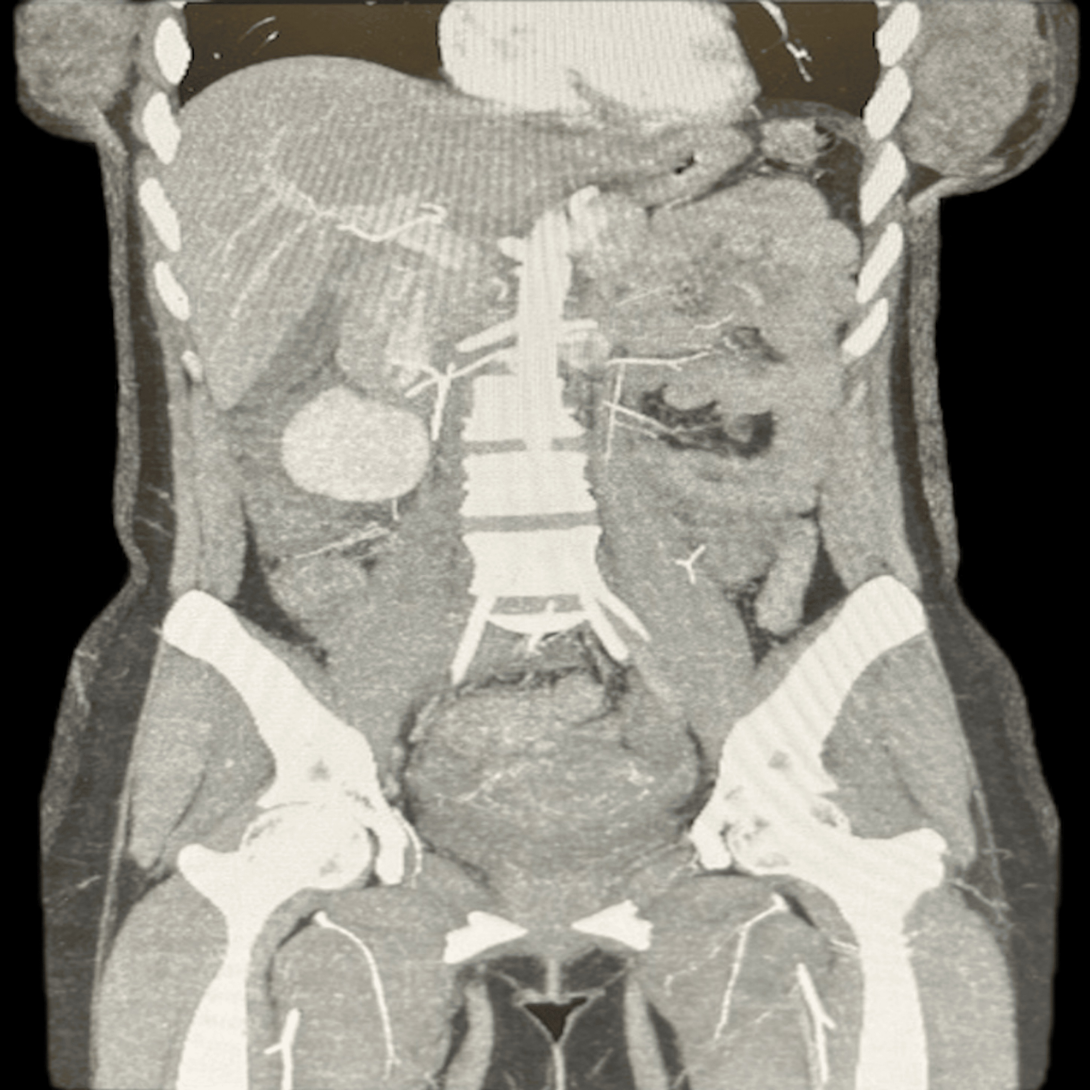
A repeat CT scan taken seven days from the last procedure found no signs of extravasation and showed decreasing fluid collection.

A repeat CT scan (Figure 3) taken seven days from the last procedure found no signs of extravasation and showed decreasing fluid collection. An X-ray with gastrografin showed no contrast extravasation. The patient’s pain continued to improve. She was restarted on a liquid diet eight days after her last procedure and advanced to a regular diet by day nine. She was ultimately discharged 21 days after her initial admission. During her follow-up, she reported doing well, did not endorse any leg pain or swelling, and stated her abdominal pain had resolved.

The patient’s hypercoagulable state initially began during her pregnancy, where she suffered from multiple thrombotic events leading to the subsequent loss of pregnancy. Hypercoagulable workup did not demonstrate any abnormal findings in acquired versus genetic factors. The patient will ultimately require an extensive outpatient workup to determine the etiology behind her hypercoagulability.

## Discussion

Duodenal perforation is a potential complication in the insertion or removal of IVC filters. Although these devices play a crucial role in providing PE prophylaxis in patients with absolute contraindications to anticoagulation therapy, they carry their own risks. In patients who are known to have an IVC filter placement and begin to experience abdominal pain, a high clinical suspicion of IVC filter perforation of an organ should be raised, as seen in our patient who developed a retroperitoneal hematoma following the removal of her IVC filter [6]. Since removing these filters also carries a risk of blood vessel injury, the presence of pneumoperitoneum within the abdomen was a critical finding in identifying the true etiology of the injury [7]. No surgical intervention was indicated at the time, as the patient remained hemodynamically stable and showed no signs of decompensation.

Currently, less than half of all IVC filters that are placed are subsequently removed [8]. Although the actual placement and removal of IVC filters carry their own dangers, leaving them in place for an extended period also increases the likelihood of patients experiencing adverse effects. In our patient’s particular case, her filter was left in place for over five months, which put her in the timeframe for removal after 30 days, where we note a higher incidence of complications [2]. It is then possible to theorise that her delayed retrieval time could have led, at least in part, to the complications observed during retrieval. Low retrieval rates of IVC filters have led to increased complications, which is why an early removal should be completed as soon as clinically indicated.

## Conclusions

IVC filter retrieval is an important component in preventing long-term complications associated with the use of these devices. Longer retrieval times have been associated with a significant number of complications, leading to decreased benefit-to-risk ratio when filters are left in place for longer. It is important to acknowledge that retrieval still carries the risk of its own complications, such as causing a perforation in surrounding structures. Nevertheless, even when dealing with complications as serious as a duodenal perforation, it is evident that patients can be conservatively managed with favorable outcomes.
